# Four years’ monitoring of *in vitro* sensitivity and candidate molecular markers of resistance of *Plasmodium falciparum* to artesunate-mefloquine combination in the Thai-Myanmar border

**DOI:** 10.1186/1475-2875-13-23

**Published:** 2014-01-15

**Authors:** Papichaya Phompradit, Poonuch Muhamad, Raewadee Wisedpanichkij, Wanna Chaijaroenkul, Kesara Na-Bangchang

**Affiliations:** 1 Chulabhorn International College of Medicine, Thammasat University (Rangsit Campus), Patumthani 12121, Thailand; 2 Thailand Centre of Excellence on Drug Discovery and Development, Thammasat University (Rangsit campus), Patumthani 12121, Thailand; 3 Division of Haematology, Faculty of Medicine, Ramathibodi Hospital, Mahidol University, Bangkok 10400, Thailand

**Keywords:** *Plasmodium falciparum*, Artesunate, Mefloquine, *pfmdr1*, *pfmrp1*, *pfatp6*, *pfcrt*, Gene polymorphisms

## Abstract

**Background:**

The decline in efficacy of artesunate (AS) and mefloquine (MQ) is now the major concern in areas along the Thai-Cambodian and Thai-Myanmar borders.

**Methods:**

The correlation between polymorphisms of *pfatp6*, *pfcrt, pfmdr1* and *pfmrp1* genes and *in vitro* sensitivity of *Plasmodium falciparum* isolates to the artemisinin-based combination therapy (ACT) components AS and MQ, including the previously used first-line anti-malarial drugs chloroquine (CQ) and quinine (QN) were investigated in a total of 119 *P. falciparum* isolates collected from patients with uncomplicated *P. falciparum* infection during 2006–2009.

**Results:**

Reduced *in vitro* parasite sensitivity to AS [median (95% CI) IC_50_ 3.4 (3.1-3.7) nM] was found in 42% of the isolates, whereas resistance to MQ [median (95% CI) IC_50_ 54.1 (46.8-61.4) nM] accounted for 58% of the isolates. Amplification of *pfmdr1* gene was strongly associated with a decline in susceptibility of *P. falciparum* isolates to AS, MQ and QN. Significant difference in IC_50_ values of AS, MQ and QN was observed among isolates carrying one, two, three, and ≥ four gene copies [median (95% CI) AS IC_50_: 1.6 (1.3-1.9), 1.8 (1.1-2.5), 2.9 (2.1-3.7) and 3.1 (2.5-3.7) nM, respectively; MQ IC_50_: 19.2 (15.8-22.6), 37.8 (10.7-64.8), 55.3 (47.7-62.9) and 63.6 (49.2-78.0) nM, respectively; and QN IC_50_: 183.0 (139.9-226.4), 256.4 (83.7-249.1), 329.5 (206.6-425.5) and 420.0 (475.2-475.6) nM, respectively]. The prevalence of isolates which were resistant to QN was reduced from 21.4% during the period 2006–2007 to 6.3% during the period 2008–2009. *Pfmdr1* 86Y was found to be associated with increased susceptibility of the parasite to MQ and QN. *Pfmdr1* 1034C was associated with decreased susceptibility to QN. *Pfmrp1* 191Y and 1390I were associated with increased susceptibility to CQ and QN, respectively.

**Conclusion:**

High prevalence of CQ and MQ-resistant *P. falciparum* isolates was observed during the four-year observation period (2006–2009). AS sensitivity was declined, while QN sensitivity was improved. *Pfmdr1* and *pfmrp1* appear to be the key genes that modulate multidrug resistance in *P. falciparum*.

## Background

Southeast Asia, particularly the Thai-Cambodian border, is one of the malaria-endemic region where multidrug-resistant *Plasmodium falciparum* malaria has been reported [1]. In the 1960s and 1970s, chloroquine (CQ) resistance spread throughout the region and subsequently, in the 1980s, resistance to sulphadoxine and pyrimethamine was reported [2]. In 1984, mefloquine (MQ) was firstly introduced for clinical use as first-line treatment for uncomplicated multidrug-resistant *P. falciparum* malaria in Thailand, but MQ resistance was rapidly developed four years after its implementation. Following the decline in clinical efficacy of MQ, the artemisinin-based combination therapy (ACT) using the artesunate-mefloquine combination was introduced as first-line reatment in 1994 [3]. Cure rate was improved to over 90% and the incidence of *P. falciparum* malaria was markedly reduced [4,5]. Artemisinin resistance however, initially occurred during 2006–2007 in areas along the Thai-Cambodian border [6]. With regard to the Thai-Myanmar border, treatment failure following ACT has been increasing especially in Tak province [7]. Studies during 2008–2009 showed a marked decline in the 42-day cure rate from 99.2 to 72.58% [8,9]. It is unclear whether artemisinin resistance has spread from the eastern to the western border of the country. Monitoring and identifying factors contributing to this low cure rate is necessary for the country’s future perspective of malaria control policy. Applying genetic analysis as a tool for detecting the genetic change of malaria parasite genes that have been shown to link with the decline in efficacy of artesunate (AS) and MQ; i.e., *pfmdr1*[10], *pfatp6*[11] and *pfmrp1*[12-14], in association with the *in vitro* sensitivity of the parasite to both combination partners, would help to detect early changes in *P. falciparum* sensitivity to this combination therapy.

In the present study, the association between the polymorphisms of *pfatp6*, *pfcrt, pfmdr1,* and *pfmrp1* genes and *in vitro* sensitivity of *P. falciparum* isolates to AS, MQ, as well as CQ and quinine (QN) was investigated in *P. falciparum* isolates collected from the Thai-Myanmar border. In addition, the change in parasite genetic patterns and *in vitro* sensitivity over the period 2006–2009 was also examined.

## Methods

### Blood samples

**Figure 1:**
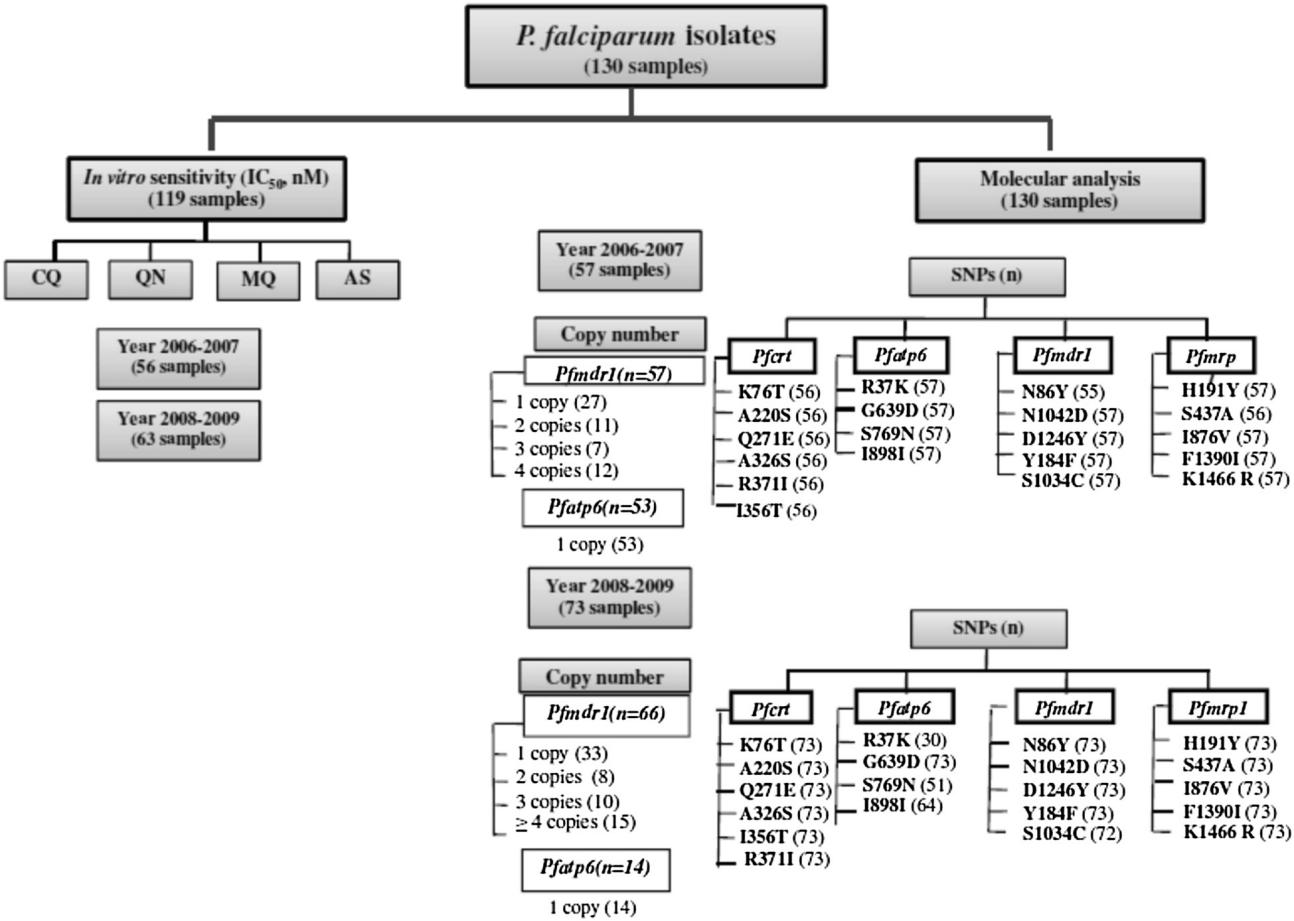
**Frameworks of *in vitro *sensitivity and molecular analysis in *Plasmodium falciparum *isolates collected during 2006–2009.** The number in parentheses signify the number of isolates included in the analysis.

A total of 130 *P. falciparum* isolates were collected from patients with uncomplicated *P. falciparum* infection prior to treatment with a three-day combination regimen (25 mg/kg body weight MQ and 12 mg/kg body weight AS) for investigation of the polymorphisms of candidate molecular markers of anti-malarial drug resistance and *in vitro* parasite sensitivity. The study was conducted at Mae Tao clinic, Mae Sot District, Tak Province, Thailand during 2006–2009. Fifty-seven and 73 isolates were collected from patients during 2006–2007 and 2008–2009, respectively (Figure 1). The study protocol was approved by the Ethics Committee of the Ministry of Public Health of Thailand and written informed consents for study participations were obtained from all patients before study.

### Culture system and *in vitro* sensitivity assay

All parasite isolates were adapted to continuous culture according to the method of [15] with modification. The laboratory-adapted 3D7 (CQ-sensitive) and K1 (CQ-resistant) *P. falciparum* were used as control parasite clones. Susceptibility of *P. falciparum* isolates to AS, MQ, CQ and QN was investigated using SYBR Green I assay [16,17]. Highly synchronous ring stage parasites were used in each assay. An aliquot of parasite inoculum (50 μl) with 2% parasitaemia and 1% haematocrit was added into each well of the 96-well microtitre plate. The plate was pre-dosed with each anti-malarial drug at eight final concentrations as follows: AS: 0.39, 0.78, 1.56, 3.13, 6.25, 12.5, 25, and 50 nM; MQ: 3.13, 6.25, 12.5, 25, 50, 100, 200, and 400 nM; CQ (3.13, 7.81, 15.63, 31.25, 62.5, 125, 250, and 500 nM); and QN: 7.81, 15.63, 31.25, 62.5, 125, 250, 500, and 1,000 nM. Standard compounds of all anti-malarial drugs were purchased from Sigma-Aldrich Co (St Louis, MO, USA). All were stored at −20°C as 10 mM stock solutions in 50% ethanol. The experiments were repeated three times, triplicate for each experiment. IC_50_ value (concentration that inhibits parasite growth by 50%) was used as an indicator for anti-malarial susceptibility and was determined from a log-dose response analysis using the CalcuSyn™ computer program (Biosoft, Cambridge, UK). The criterion used for susceptibility of the parasite isolates to CQ was as follow: sensitive (S: IC_50_ <25 nM), moderately resistant (MR: 25 ≤ IC_50_ <100 nM), and highly resistant (HR: IC_50_ ≥100 nM). QN susceptibility was categorized into two levels, i.e., S (IC_50_ <500 nM), and resistant (R: IC_50_ ≥500 nM). MQ susceptibility was categorized into two levels, i.e., “sensitive” (IC_50_ ≤24 nM) and “resistant” (IC_50_ >24 nM). For AS, the susceptibility was classified as “sensitive” (IC_50_ ≤ upper limit of 95% CI of the median IC_50_) and “declined sensitivity” (IC_50_ > upper limit of 95% confidence interval (CI) of the median IC_50_) [18].

### Investigation of polymorphisms of *pfcrt*, *pfmdr1*, *pfatp6,* and *pfmrp1* using PCR-RFLP

Genomic DNA was extracted from all samples (culture-adapted *P. falciparum*; Figure 1) using chelex resin modified technique [19]. Prior to being used as a DNA template, concentration of the malaria genomic DNA was determined by spectrophotometry (Nanodrop^TM^, Thermo fisher Scientific, Massachusetts, USA).

Previously published nested and PCR-RFLP methods were employed to detect the polymorphisms of *pfcrt* gene at amino acid residues 76, 220, 271, 326, 356, and 371 [20]; *pfmdr1* gene at amino acid residues 86, 184, 1034, 1042, and 1246 [21,22]; *pfatp6* gene at amino acid residues 37, 693, 769, 898 [23]; *pfmrp1* gene at amino acid residues 191 and 437 [24]; and *pfmrp1* gene at amino acid residues 876, 1390 and 1466 [25].

### Detection of *pfatp6* and *pfmdr1* gene copy number by SYBR Green I real-time PCR

*Pfatp6 and pfmdr1* gene copy number in all samples (Figure 1) was determined by SYBR Green I real-time PCR (iCycler™, Bio-Rad, California, USA) using the default thermocycler program: 10min of pre-incubation at 95°C, followed by 40 cycles for 15 sec at 95°C and 1 min at 60°C. The oligonucleotide primers used were those previously designed by Ferreira *et al.*[26] with modification. Individual real-time PCR reaction was carried out in a 25 μl reaction volume in a 96-well plate containing 2 μl of DNA (50 ng), 1 μM each of sense and antisense primer and 12.5 μl of Platinum™ PCR SuperMix (Invitrogen, California, USA).

The 2^-ΔΔCt^ method of relative quantification was adapted to estimate copy number in *P. falciparum* genes. The genomic DNA extracted from *P. falciparum* 3D7 clone known to harbour a single copy of each gene was used as a calibrator, while Pf-β-actin 1 served as the house-keeping gene in all experiments. Dd2 genomic DNA carrying four copies of *pfmdr1* was used as a second calibrator. The threshold cycle (Ct) was determined as the increase in reporter signal, which was first detected above baseline. Results were analysed by a comparative Ct method based on the assumption that the target (*pfatp6* and *pfmdr1*) and reference (pf-β-actin 1) genes were amplified with the same efficiency within an appropriate range of DNA concentrations.

The ΔΔCt calculation for the relative quantification of target was as follow: ΔΔCt = (Ct, target gene − Ct, Pf-β- actin1)_x_ − (Ct, target gene − Ct, Pf-β-actin1)_y_, where x represents unknown sample and y represents *P. falciparum* 3D7 clone. Results for each sample was expressed as an *N*-fold change in χ target gene copies, normalized to Pf-β-actin-1 relative to the copy number of the target gene in *P. falciparum* 3D7 clone, according to the following equation: amount of target = 2^-ΔΔCt^. A minimum of two experiments were carried out for each gene and each sample. In each experiment, each individual sample was analysed in duplicate wells and the Ct of each well was recorded at the end of the reaction.

### Statistical analysis

The association between *in vitro* sensitivity of *P. falciparum* isolates and polymorphisms of *pfcrt, pfmdr1*, *pfmrp1,* and *pfatp6* was analysed using Chi-square and Mann–Whitney U tests. Correlation between the two quantitative variables was evaluated using Spearman correlation test. The qualitative variables are summarized as proportions and percentages and the quantitative variables are summarized as median (95% CI) values. Statistical significance level was set at *α* = 0.05 for all tests (SPSS version 15; SPSS, Chicago, Illinois, USA).

## Results

### *In vitro* sensitivity of *Plasmodium falciparum* isolates

**Figure 2:**
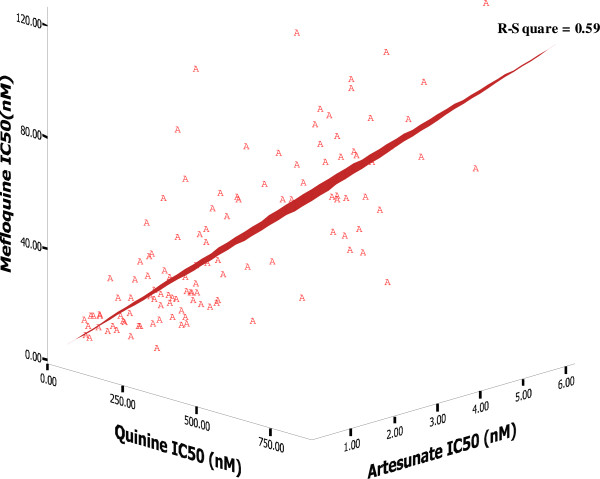
**The three-dimensional scatter diagram and the regression line representing relationships between IC**_**50 **_**(nM) values of mefloquine (MQ), quinine (QN) and artesunate (AS).**

*In vitro* sensitivity to AS, MQ, CQ, and QN was successfully evaluated in a total of 119 *P. falciparum* isolates (Figure 1 and Table 1), 56 and 63 isolates collected during 2006–2007 and 2008–2009, respectively (Figure 1 and Table 2). Isolates with declined sensitivity to AS [median (95% CI) 3.4 (3.1-3.7) nM] were observed in 42% (50 isolates). Fifty-eight per cent (69 isolates) were identified as MQ-resistant [median (95% CI) = 54.1 (46.8-61.4) nM]. Almost all (99.2%, 118 isolates) were identified as moderately and highly CQ-resistant; only one (0.8%) isolate was identified as CQ-sensitive (IC_50_ values = 9.6 nM). About 13% (16 isolates) were identified as QN-resistant [median (95% CI) = 648.9 (621.2-676.5) nM]. A positive significant correlation was observed between the IC_50_ values of MQ and AS (*r* = +0.662; *p* <0.001), MQ and QN (*r* = +0.750; *p* <0.001), and AS and QN (*r* = +0.625; *p* <0.001) (Figure 2). There was no significant difference in the IC_50_ values of all drugs between isolates collected during the two periods. However, the prevalence of QN-resistant isolates collected during 2008–2009 was significantly lower than during 2006–2007 (*p* = 0.016).

**Table 1:** ***In vitro *** **sensitivity of *Plasmodium falciparum *** **isolates (represented by IC**_**50 **_**values) to chloroquine (CQ), quinine (QN), mefloquine (MQ), and artesunate (AS) and their sensitivity classification**

**Drug**	**Sensitivity classification**	**% (n)**	**IC**_**50**_**, nM**
AS	All	100 (119)	2.0 (1.7–2.3)
	Sensitive (IC_50_ ≤2.3 nM)	58.0 (69)	1.4 (1.8–2.8)
	Declined sensitivity (IC_50_ >2.3 nM)	42.0 (50)	3.4 (3.1–3.7)
MQ	All	100 (119)	30.1 (21.8–33.3)
	Sensitive	42.0 (50)	15.2 (12.8–17.6)
	Resistant	58.0 (69)	54.1 (46.8–61.4)
CQ	All	100 (119)	72.3 (67.3–77.3)
	Sensitive	0.8 (1)	9.6
	Moderate resistant	73.1 (87)	66.6 (63.5–69.7)
	Highly resistant	26.1 (31)	131.6 (118.9–144.4)
QN	All	100 (119)	231.7 (192.2–271.2)
	Sensitive	86.6 (103)	222.6 (195.5–249.8)
	Resistant	13.4 (16)	648.9 (621.2–676.5)

Data presented as median (95% CI) and percentage (number).

**Table 2:** **In vitro sensitivity of *Plasmodium falciparum *** **isolates collected during 2006–2007 and 2008–2009 to chloroquine (CQ), quinine (QN), mefloquine (MQ), and artesunate (AS) and their sensitivity classification**

**Drug**	**Sensitivity classification**	**Year 2006–2007**		**Year 2008–2009**	
		**% (n)**	**IC**_**50**_**, nM**	**% (n)**	**IC**_**50**_**, nM**
AS	All	100 (56)	2.0 (1.5–2.4)	100 (63)	2.0 (1.5–2.5)
	Sensitive	60.7 (34)	1.4 (1.3–1.6)	58.7 (37)	1.4 (1.1–1.8)
	Declined sensitivity	39.3 (22)	3.1 (2.8–3.3)	41.3 (26)	3.7 (3.2–4.2)
MQ	All	100 (56)	30.4 (18.0–42.7)	100 (63)	28.8 (16.7–41.0)
	Sensitive	42.9 (24)	13.8 (11.3–16.3)	41.3 (26)	16.2 (13.2–19.2)
	Resistant	57.1 (32)	51.5 (40.3–62.7)	58.3 (37)	58.7 (49.3–68.1)
CQ	All	100 (56)	69.9 (62.8–77.1)	100 (63)	72.3 (65.4–79.2)
	Sensitive	1.8 (1)	9.6	–	–
	Moderate resistant	76.8 (43)	65.9 (59.9–72.0)	69.8 (44)	66.6 (62.3–70.8)
	Highly resistant	21.4 (12)	124.3 (108.1–140.6)	30.2 (19)	132.3 (68.1–196.5)
QN	All	100 (56)	236.3 (186.2–286.4)	100 (63)	230.5 (164.8–296.2)
	Sensitive	78.6 (44)	223.0 (178.6–267.3)	93.7 (59)	214.9 (150.4–279.4)
	Resistant	21.4 (12)	648.9 (606.1–691.6)	6.3 (4)^**^	593.6 (491.0–696.2)

Data presented as median IC_50_ (95% CI) and percentage (number).

^**^Significant lower proportion than isolates collected from 2006–2007 (*p* = 0.016, Chi–square test).

### Candidate molecular markers of antimalarial drug resistance in *Plasmodium falciparum* isolates

#### *Pfatp6* mutation

No mutation in *pfatp6* at the target amino acid residues 37, 639 and 769 was found in any isolate (0/87, 0/130, and 0/108 isolate, respectively), whereas the mutation at 898 was detected in six out of 121 (5.0%) isolates. Almost all isolates collected during 2006–2007 (100%) and 2008–2009 (93.7%) carried wild type *pfatp6* at the target amino acid residues 37, 639, 769, and 898. There was a significant difference in the prevalence of isolates collected during 2006–2007 (0%, 0/57) and 2008–2009 (9.4%, 6/64) with regard to the mutation at the amino acid residue 898 (*p =* 0.018).

#### *Pfcrt* mutation

Almost all isolates carried mutated *pfcrt* gene. The prevalence of gene mutation at codons 76T, 220S, 271E, 326S, and 371I was 99.2% (128/129), while the mutation at the codon 356T was 98.4% (127/129).

#### *Pfmdr1* mutation

No mutation in *pfmdr1* at the codons 1042D and 1246Y was observed (0/130 and 0/130) in any isolate collected during the two investigation periods. The prevalence of *pfmdr1* wild type at the target amino acid residue 86 was significantly higher in isolates collected during 2008–2009 (100%, 73/73) compared with 2006–2007 (94.5%, 52/55) (*p =* 0.043). The prevalence of *pfmdr1* wild type at the target amino acid residue 184 in isolates collected during 2006–2007 and 2008–2009 was 100% (57/57) and 94.5% (69/73), respectively. For the target amino acid residue 1034, mutant, heterozygous mutant and wild type isolates were detected in 15.8% (14/57), 56.6% (34/57) and 24.6% (9/57) of the isolates collected during 2006–2007, respectively. The prevalence of the mutant and heterozygous mutant at the same amino acid target was detected in 98.6 and 1.4% of the isolates collected during 2008–2009, respectively. No wild type isolate was found at this target amino acid. The prevalence of gene mutation at the amino acid residue 1034 was significantly higher in isolates collected between 2008–2009 compared with 2006–2007 (*p* < 0.001).

#### *Pfmrp1* mutation

No mutation in *pfmrp1* at the codon 1466R was observed (0/130) in any isolate collected during both periods. The mutations at amino acid residues 191, 437, 876, and 1390 were detected in 86.9% (113/130), 89.1% (115/129), 74.6% (97/130) and 26.2% (34/130), respectively. The prevalence of isolates with mutations at amino acid residues 191 and 437 collected during 2006–2007 *vs* 2008–2009 were 84.2% (48/57) *vs* 89.0% (65/73) and 85.7% (48/56) *vs* 91.8% (68/73), respectively. The prevalence of isolates with mutations at amino acid residues 876 (*p* < 0.001) and 1390 (*p* = 0.037) was significantly higher in 2008–2009 (42.5%, 31/73) compared with 2006–2007 (5.3%, 3/57).

#### *Pfmdr1* copy number

Isolates which carried one, two, three, four, five, six and eight gene copies were found in 48.8% (60/123), 15.4% (19/123), 13.8% (17/123), 17.1% (21/123), 0.8% (1/123), 3.3% (4/123), and 0.8% (1/123), respectively. For the period 2006–2007, the isolates which carried one, two, three and four gene copies were found in 47.4% (27/57), 19.3% (11/57), 12.3% (7/57) and 21.1% (12/57), respectively. About 50% (33/66), 12.1% (8/66), 15.2% (10/66), 13.6% (9/66), 1.5% (1/66), 6.1% (4/66), and 1.5% (1/66) of the isolates collected during 2007–2008 carried one, two, three, four, five, six and eight gene copies, respectively. There was no significant difference in the prevalence of *pfmdr1* gene copy in isolates collected during the two periods.

#### *Pfatp6* copy number

All of the 67 isolates under the analysis from the two periods carried only one gene copy.

### Association between polymorphisms of candidate molecular markers of anti-malarial drug resistance and *in vitro* sensitivity of *Plasmodium falciparum* isolates

**Figure 3:**
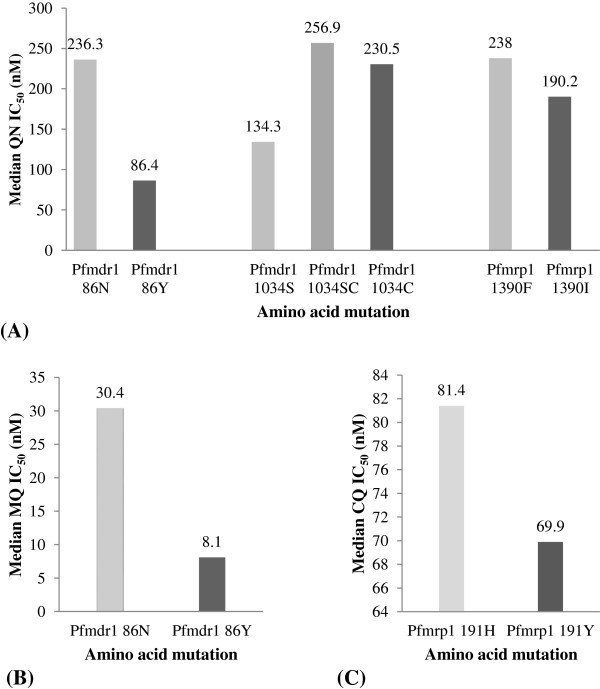
**The relationship between median IC**_**50 **_**values of quinine (QN), mefloquine (MQ) and chloroquine (CQ) and the mutations of *pfmdr1 *and *pfmrp1 *gene in *Plasmodium falciparum *isolates.** Association between **(A)** QN IC_50_ and *pfmdr1* N86Y (*p* = 0.015, Mann–Whitney U test), S1034C (*p* = 0.018, Kruskal-Wallis test) and *pfmrp1* F1390I mutation (*p* = 0.039, Mann–Whitney U test); **(B)** MQ IC_50_ and *pfmdr1* N86Y mutation (*p* = 0.005, Mann–Whitney U test); and **(C)** CQ IC_50_ and *pfmrp1* H191Y mutation (*p* = 0.008, Mann–Whitney U test).

The association between polymorphisms of candidate molecular markers of anti-malarial drug resistance and *in vitro* sensitivity to anti-malarial drugs was investigated in 119 matched-paired *P. falciparum* isolates (Figure 3).

#### *Pfatp6* mutation and *pfatp6* copy number

No significant association was observed between *pfatp6* mutation including *pfatp6* copy number and *in vitro* sensitivity of *P. falciparum* isolates to all drugs.

#### *Pfcrt* mutation

Significant association was observed between CQ resistance and *pfcrt* mutations at codons 76T, 220S, 271E, 326S, and 371I. Only one CQ sensitive isolate (IC_50_ 9.6 nM) carried wild type genotype at codons 76, 220, 271, 326, and 371 (*p* = 0.007).

#### *Pfmdr1* mutation

The IC_50_ values of MQ and QN were significantly lower in the three (out of 117) isolates with 86Y mutation compared with those carried wild type (86 N) genotype [median (95% CI) MQ IC_50_: 30.4 (21.9-38.8) *vs* 8.1 (3.4-9.1) nM, *p* = 0.005; median (95% CI) QN IC_50_: 236.3 (195.7-276.9) *vs* 86.4 (32.2-119.1) nM, *p* = 0.015). The isolates carrying 1034S had significantly lower QN IC_50_ than that carrying 1034SC [IC_50_ (95%CI) 134.3 (46.3-222.6) *vs* 230.5(168.3-292.7) nM, *p* = 0.007].

#### *Pfmrp1* mutation

CQ IC_50_ was significantly lower in isolates carrying 191Y compared with that carrying wild type (H) genotype [median (95% CI) IC_50_ 81.4 (58.2-104.7) and 69.9 (65.0-74.9) nM, respectively; *p* = 0.008]. The QN IC_50_ was significantly lower in isolates carrying 1390I compared with that carrying wild type 1390F genotype [median (95% CI) QN IC_50_: 238.0 (191.0-285.1) *vs* 190.2 (100.7-279.8), *p* = 0.039].

#### *Pfmdr1* copy number

**Figure 4:**
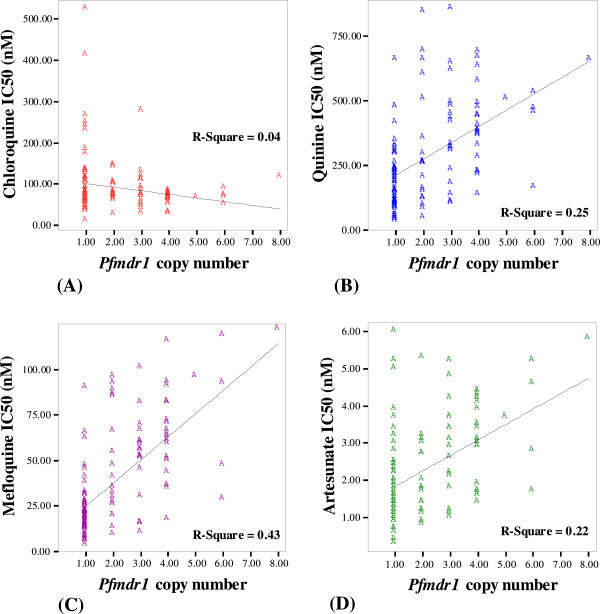
**Correlation between *pfmdr1 *** **copy number and *in vitro *** **sensitivity of *Plasmodium falciparum i*** **solates to (A) chloroquine (CQ), (B) quinine (QN), (C) mefloquine (MQ), and, (D) artesunate (AS).**

A marked difference in IC_50_ values of AS, MQ and QN was observed among isolates carrying one, two, three, and ≥ four gene copies [median (95% CI) AS IC_50_: 1.6 (1.3-1.9), 1.8 (1.1-2.5), 2.9 (2.1-3.7) and 3.1 (2.5-3.7) nM, *p* < 0.001; MQ IC_50_: 19.2 (15.8-22.6), 37.8 (10.7-64.8), 55.3 (47.7-62.9) and 63.6 (49.2-78.0) nM, *p* < 0.001; and QN IC_50_: 183.0 (139.9-226.4), 256.4 (83.7-249.1), 329.5 (206.6-425.5) and 420.0 (475.2-475.6) nM, respectively, *p* < 0.001]. Significant positive correlation was found between the increased *pfmdr1* copy number and *in vitro* sensitivity of *P. falciparum* isolates to AS (*r* = +0.47, *p* < 0.001), MQ (*r* = +0.65, *p* < 0.001), and QN (*r* = +0.50, *p* < 0.001), while a significant negative correlation was found with CQ (*r* = −0.19, *p* = 0.038) (Figure 4).

## Discussion

The present study investigated the relationship between *P. falciparum* gene polymorphisms (*pfatp6*, *pfcrt, pfmdr1,* and *pfmrp1*) and *in vitro* sensitivity of *P. falciparum* isolates to the ACT combination partners AS and MQ, including CQ and QN in a multidrug-resistant area along the Thai-Myanmar border during the two periods (2006–2007 and 2008–2009). Results suggest a significant change in the prevalence and pattern of *pfatp6*, *pfmdr1* and *pfmrp1* gene polymorphisms with no change in the *in vitro* sensitivity profiles of the parasite during the two periods. Decline in sensitivity of *P. falciparum* to MQ was continuously reported during 1991–1994 when MQ monotherapy was employed in Thailand [27]. However, until a decade after the switch of first-line treatment to AS-MQ combination, high prevalence and intensity of MQ resistance was reported (32%, 46% and 58% during 1998–2005 [28], 2007–2008 [29], and 2006–2009 (the current study), respectively). While evidence of artemisinin resistance was documented in Western Cambodia during the period 2006–2007 [30,31], sensitivity of *P. falciparum* isolates in this area to AS during the same period was generally considered satisfactory [18,29,32,33]. Subsequent reports on a decline in AS sensitivity [7,9] nevertheless, has created a major concern on the future use of AS in combination therapy. Without definite criteria for defining artemisinin resistance, the upper limit of 95% CI of median IC_50_ value of AS in isolates collected from all patients (2.3 nM) was used as a cut-off level of AS resistance in the present study. Based on this cut-off level, approximately 42% of the isolates were classified as declined sensitivity to AS. The prevalence of isolates with MQ resistance and declined sensitivity to AS observed in the present study were similar to that previously reported (58 *vs* 57.6% and 42 *vs* 36.7%, respectively) [34]. This cut-off criteria for defining AS resistance (the upper limit of 95% CI of median IC_50_ value of AS) was slightly lower in the current study (2.3 nM) compared with the previous study (2.8 nM) [34]. The sensitivity to AS (IC_50_: 2.0 *vs* 1.7 nM) and MQ (30.1 *vs* 34.0) were comparable with that reported in the isolates collected from Cambodia during the same period [35].

It was noted for the improvement of parasite sensitivity in this area to CQ and QN since the introduction of AS-MQ combination in Thailand in 1995. There was even one isolate that was sensitive to CQ. In 1991–1992 and 1994, the degree of CQ resistance in the country was high [median (95% CI) IC_50_ 193.2 (148.24-251.85) and 157.0 (124.11-198.73) nM, respectively] [27], but was improved during the 1997, 1999 and 2004–2009 [median (95% CI) IC_50_: 157.0 (128.0–193.0), 120.5 (105.7–137.3) and 70.0 (10.0-183.0) nM, respectively] [18,36,37]. Molecular analysis in either laboratory or field *P. falciparum* isolates demonstrated the strong linkage between CQ resistance and *pfcrt* gene mutations [20,22,38]. Susceptibility of the parasite to CQ was also shown in this study to link with *pfmrp1* polymorphism, of which the 191Y mutation resulted in increased susceptibility of the parasite to CQ. For QN, a decline in parasite sensitivity to the drug was obviously observed during 1991–1992 and 1994 [mean (95% CI) IC_50_ 576.2 (460.51-720.99) and 403.9 (330.50-493.54) nM, respectively], but was improved during 1997–2008 [18,36,37,39]. A total of 16 isolates were identified as QN resistance, 12 and four isolates collected during 2006–2007 and 2008–2009, respectively.

*Pfmdr1* and *pfmrp1* appear to be the key genes involved in resistance of *P. falciparum* to the commonly used anti-malarial drugs. The *pfmdr1* 86Y mutation leads to increase in susceptibility of the parasite to MQ compared with wild type genotype (IC_50_ of 8.1 and 30.4 nM, respectively) as well as the structurally related anti-malarial QN (IC_50_ of 86.4 and 236.3 nM). The results showed low prevalence of 184F allele in parasite isolates collected during the investigation period. The 184F allele was reported to be associated with increased IC_50_ of MQ. High prevalence of (~86%) of the 184F allele was reported in western Cambodia where the level of MQ resistance was significant. On the other hand, the prevalence of 184F allele in eastern Cambodia was low (~32%), which was also correlated with the reduced level of MQ resistance in this region [40]. Molecular analysis in the current study revealed an obvious involvement of *pfmdr1* and *pfmrp1* genes with QN-resistant *P. falciparum*[13,14,21,41]. The *pfmdr1* 86Y and *pfmrp1* 1390I mutations resulted in an improvement of sensitivity of the parasite to QN compared with the wild type isolates [(86.4 *vs* 236.3 nM) and (190.2 *vs* 238.0 nM), respectively]. On the other hand, the mutation and heterozygous mutation of *pfmdr1* 1034C resulted in the decreased susceptibility of the parasite to QN (median IC_50_ of 230.5, 256.9 and 190.3 nM for mutated, heterozygous mutated and wild type genotypes, respectively). Interestingly, the prevalence of *pfmrp1* 1390 (I) in parasite isolates collected during 2008–2009 was higher than that collected during 2006–2007.

With regard to the *pfatp6* polymorphisms, there were no mutations found at codons 37, 639 and 769 in samples collected during 2006–2009. The mutation at codon 898 was found in isolates collected from 2008–2009. Although this residue is silent mutation, this observation may imply on the influence of anti-malarial drug pressure on the parasite during that period. The finding was in agreement with that reported by Jambou and colleges for the isolates in Asia including Thailand [11]. It is noted however that *in vitro* cultivation may cause the poor fitness of the mutant genotypes, which may explain the observation of almost absence of the mutation in *pfatp6* gene in this study [42].

The current results support the role of *pfmdr1* amplification in modulating the degree of AS, MQ and QN susceptibility [43,44] A trend of increasing number of gene copies and increasing IC_50_ values of AS, MQ and QN was clearly observed. Approximately 51% of the isolates carried *pfmdr1* copy number ranging from two to eight copies. The study conducted during 1995–2007, the period of which the clinical efficacy of AS-MQ combination was satisfactory, showed the increase in *pfmdr1*copy number from 30% (12/40) in 1996 to 53% (24/45) in 2006 [45]. Besides the high level of initial parasitaemia, prolongation of parasite clearance time and reduced *in vitro* parasite sensitivity to MQ, treatment failure following AS-MQ combination therapy was also associated with increased *pfmdr1* copy number. Parasite isolates collected prior to treatment from patients with recrudescent response were found to carry higher number of *pfmdr1* copies compared to those with sensitive response (mean copies of 2.7 *vs* 1.9). In addition, isolates collected on the day of recrudescence carried higher number of gene copies than the corresponding day 0 samples (mean copies 3.5 *vs* 2.6) [35]. In a previous study, about 70% of isolates collected from patients with recrudescence response before AS-MQ treatment was shown to carry more than 1 *pfmdr1* copy number and increase in *pfmdr1* copy number was associated with reduced parasite sensitivity to AS, or resistant to MQ, or both [34]. Although the artemisinin resistance has not been clearly defined in this study, results suggest that both the parasite (reduced *in vitro* sensitivity and increased *pfmdr1* copy number) and host (pharmacokinetic variability) factors might contribute to artemisinin resistance [9,46].

## Conclusions

Based on results of the current observation on *in vitro* sensitivity and candidate molecular markers of resistance, it is concluded that high prevalence of MQ resistance still remained during the four years’ observation period (2006–2009). In addition, sensitivity of the parasite to AS appeared to be declining. *Pfmdr1* gene copy number is the key molecular marker of resistance of *P. falciparum* isolates in this area to AS-MQ combination therapy.

## Competing interests

The authors declare that they have no competing interests.

## Authors’ contributions

KN was involved in providing the conception, design of the study and revised the manuscript critically for intellectual content and approved the final version of the manuscript. PP, PM and RW performed the investigation on the polymorphisms of *pfatp6*, *pfcrt, pfmdr1 and pfmrp1* genes and *in vitro* sensitivity test. PP and WC performed data analysis and interpretation. PP drafted the manuscript. All authors read and approved the final manuscript.
